# Years of life lost due to deaths of despair and COVID-19 in the United States in 2020: patterns of excess mortality by gender, race and ethnicity

**DOI:** 10.1186/s12939-023-01949-9

**Published:** 2023-08-23

**Authors:** Parker Entrup, Leon Brodsky, Candice Trimble, Stephanie Garcia, Nasra Mohamed, Megan Deaner, J. P. Martell, Julie Teater, Ayana Jordan, Jeanette M. Tetrault, O. Trent Hall

**Affiliations:** 1 Department of Psychiatry and Behavioral Health, Ohio State University Wexner Medical Center Talbot Hall, 181 Taylor Ave., Columbus, OH 43203 USA; 2 College of Medicine, the Ohio State University, Columbus, OH USA; 3 Department of General Surgery, OU-Physicians, Tulsa, OK USA; 4 Department of Psychiatry and Behavioral Sciences, University of Kansas Health System, Kansas City, KS USA; 5 Department of Population Health NYU Grossman School of Medicine, New York City, NY USA; 6 Department of Internal Medicine, Program in Addiction Medicine, Yale School of Medicine, New Haven, CT USA

**Keywords:** COVID-19, Addiction Medicine, Substance Use, Epidemiology, Mortality, Mental Health

## Abstract

**Background:**

In 2020 COVID-19 was the third leading cause of death in the United States. Increases in suicides, overdoses, and alcohol related deaths were seen—which make up deaths of despair. How deaths of despair compare to COVID-19 across racial, ethnic, and gender subpopulations is relatively unknown. Preliminary studies showed inequalities in COVID-19 mortality for Black and Hispanic Americans in the pandemic's onset. This study analyzes the racial, ethnic and gender disparities in years of life lost due to COVID-19 and deaths of despair (suicide, overdose, and alcohol deaths) in 2020.

**Methods:**

This cross-sectional study calculated and compared years of life lost (YLL) due to Deaths of Despair and COVID-19 by gender, race, and ethnicity. YLL was calculated using the CDC WONDER database to pull death records based on ICD-10 codes and the Social Security Administration Period Life Table was used to get estimated life expectancy for each subpopulation.

**Results:**

In 2020, COVID-19 caused 350,831 deaths and 4,405,699 YLL. By contrast, deaths of despair contributed to 178,598 deaths and 6,045,819 YLL. Men had more deaths and YLL than women due to COVID-19 and deaths of despair. Among White Americans and more than one race identification both had greater burden of deaths of despair YLL than COVID-19 YLL. However, for all other racial categories (Native American/Alaskan Native, Asian, Black/African American, Native Hawaiian/Pacific Islander) COVID-19 caused more YLL than deaths of despair. Also, Hispanic or Latino persons had disproportionately higher mortality across all causes: COVID-19 and all deaths of despair causes.

**Conclusions:**

This study found greater deaths of despair mortality burden and differences in burden across gender, race, and ethnicity in 2020. The results indicate the need to bolster behavioral health research, support mental health workforce development and education, increase access to evidence-based substance use treatment, and address systemic inequities and social determinants of deaths of despair and COVID-19.

## Introduction 

The COVID-19 pandemic was the third leading cause of death in the United States in 2020 [1]. Circumstances, such as isolation, changes in routine, dissolution of support systems, income disruption, and overall anxiety regarding the virus during the first year of the COVID-19 pandemic exacerbated mental health problems and exhausted behavioral health resources. Resources which are necessary for preventing deaths of despair—defined as death from alcohol and other types of substances (accidental drug overdose) and suicide [2]. The year 2020 occasioned a record increase in accidental drug overdose deaths, alongside escalated admissions to undergo treatment for alcohol withdrawal [3, 4]. It was reported that during 2020 12.2 million US adults reported suicidal ideation and 1.2 million attempted suicides [5]. Another study demonstrated that stressors related to the pandemic provoked an increase in the prevalence of psychiatric conditions [6].

Prior reports have documented racial, ethnic, and gender inequities in mortality from COVID-19 and deaths of despair. Preliminary studies showed marked inequalities in COVID-19 mortality with increased mortality for Black and Hispanic Americans in the pandemic's onset [7]. Increases in accidental overdose deaths were documented leading up to the pandemic but were seen at disproportionately higher amounts in Native American/Native Alaskan and Black American populations during the pandemic [8].

Deaths of despair differ from deaths due to COVID-19 in that deaths of despair tend to occur at relatively younger ages representing a higher burden in terms of years of life lost (YLL). YLL is a time-based mortality statistic developed by the World Health Organization Global Burden of Disease and its analysis provides important context to number of death statistics by giving greater weight to deaths among younger decedents [9]. YLL are often calculated to compare the relative mortality burden of different causes of death among disparate populations [9, 10]. Comparative YLL analysis may be used to guide resource allocation and more precisely targeted treatment and prevention efforts toward health conditions in populations with inordinate mortality burden [9, 10]. Therefore, the present work aims to assess and compare a retrospective analysis of the relative mortality burden of deaths of despair and COVID-19 by race, ethnicity, and gender in the United States in 2020.

## Methods

This cross-sectional retrospective study utilized 2020 United States of America summary death statistics from the CDC Wide-ranging Online Data for Epidemiologic Research mortality file [11]. Data were pulled directly from WONDER using underlying cause of death query and computed in a Google Sheets spreadsheet. Life expectancy by single-year age and sex was determined from the most recent Social Security Administration Period Life Table, from 2019 [12]. YLL were tabulated for males and females at each single-year age (*YLL* = *life expectancy at age at death* X *number of deaths*). As well as tabulating for ethnicity (Hispanic orLatino//Non-Hispanic orNon-Latino) and six race categories (American Indian*/Alaskan Native, Asian, Black/African American, Native Hawaiian/Pacific Islander, White, and more than one race). *In order to be more inclusive researchers will use Native American in place of American Indian for the rest of the manuscript, while less inclusive terms are used in database. Cases were identified by International Classification of Diseases, Tenth Revision (ICD-10) codes: accidental drug overdose (X40-X44), suicide (U03, X60-X84, Y87.0), Alcohol-induced causes of death (E24.4, F10, G31.2, G62.1, G72.1, I42.6, K29.2, K70, K85.2, K86.0, R78.0, X45, X65, Y15) and COVID-19 (U07.1) [11] Exemption was granted by the Ohio State University Wexner Medical Center Institutional Review Board.

## Results

**Fig. 1:**
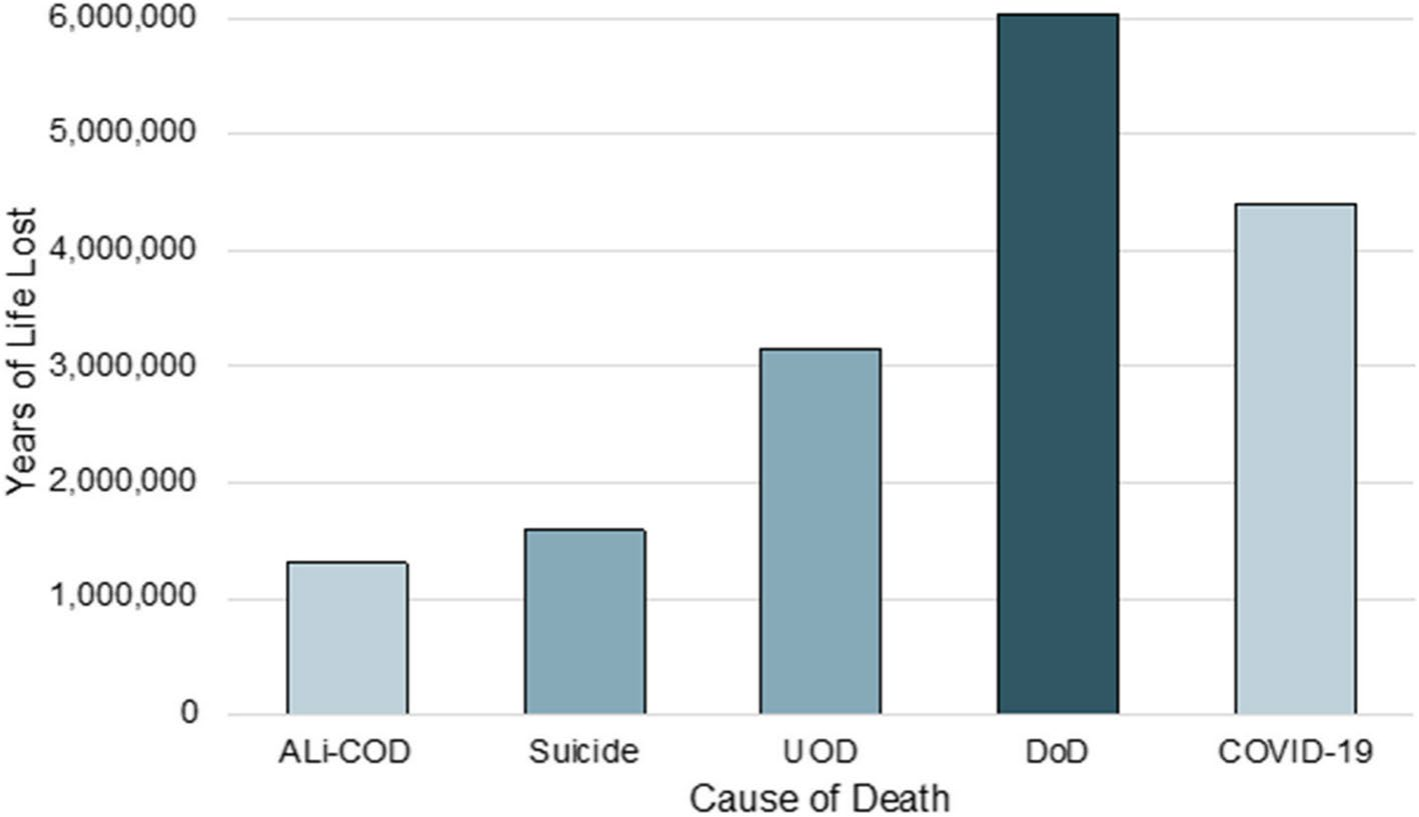
Years of life lost due to COVID-19 and deaths of despair including unintentional drug overdose, alcohol-induced causes of death, and suicide in the US in 2020

In 2020, COVID-19 caused 350,831 deaths and 4,405,698.68 YLL. By contrast, deaths of despair contributed to relatively fewer deaths at 178,598 but substantially greater mortality expressed in YLL (6,045,819.01). This discrepancy was due to differences in the average age of individuals who died of COVID-19 and deaths of despair. COVID-19 decedents were older (mean age 76.07; SD, 13.65) than deaths of despair decedents (mean age 47.17.; SD, 15.95). Despite lower number of deaths, deaths of despair caused 37.2% more YLL than COVID-19 during the study period. Figure 1 illustrates YLL due to COVID-19 and deaths of despair in 2020.

Of deaths of despair, accidental drug overdose deaths caused the greatest mortality burden (3,156,748.88 YLL); followed by suicide (1,588,508.18 YLL); and alcohol-induced causes of death (1,300,561.95 YLL). Overall, deaths of despair decedents lost an average of 33.3 years of life per person while COVID-19 decedents lost 12.6 years.

### Co-occurring COVID-19 and deaths of despair

Over 1,200 decedents were listed as having both COVID-19 and a death of despair diagnosis contributing to mortality. Accidental drug overdose co-occurred with COVID-19 causing over 518 deaths and 9,418.65 YLL. Alcohol-induced mortality was co-morbid with COVID-19 contributing to 646 deaths and 9,306.26 YLL. Finally, suicide mortality occurred with COVID-19 among 108 cases leading to 1,421.87 YLL.

### Gender, COVID-19 and deaths of despair

Gender differences were noted in deaths and YLL; men died in greater numbers and lost more years of life than women. This pattern held true for COVID-19 and all three deaths of despair sub-categories. However, the degree of gender disparity varied by cause of death. For example, men accounted for approximately 55% of COVID-19 deaths and YLL; but almost 80% of deaths and YLL from suicide. Additionally, men represented approximately 70% of deaths and YLL due to accidental drug overdose and alcohol induced causes of death.

**Fig. 2:**
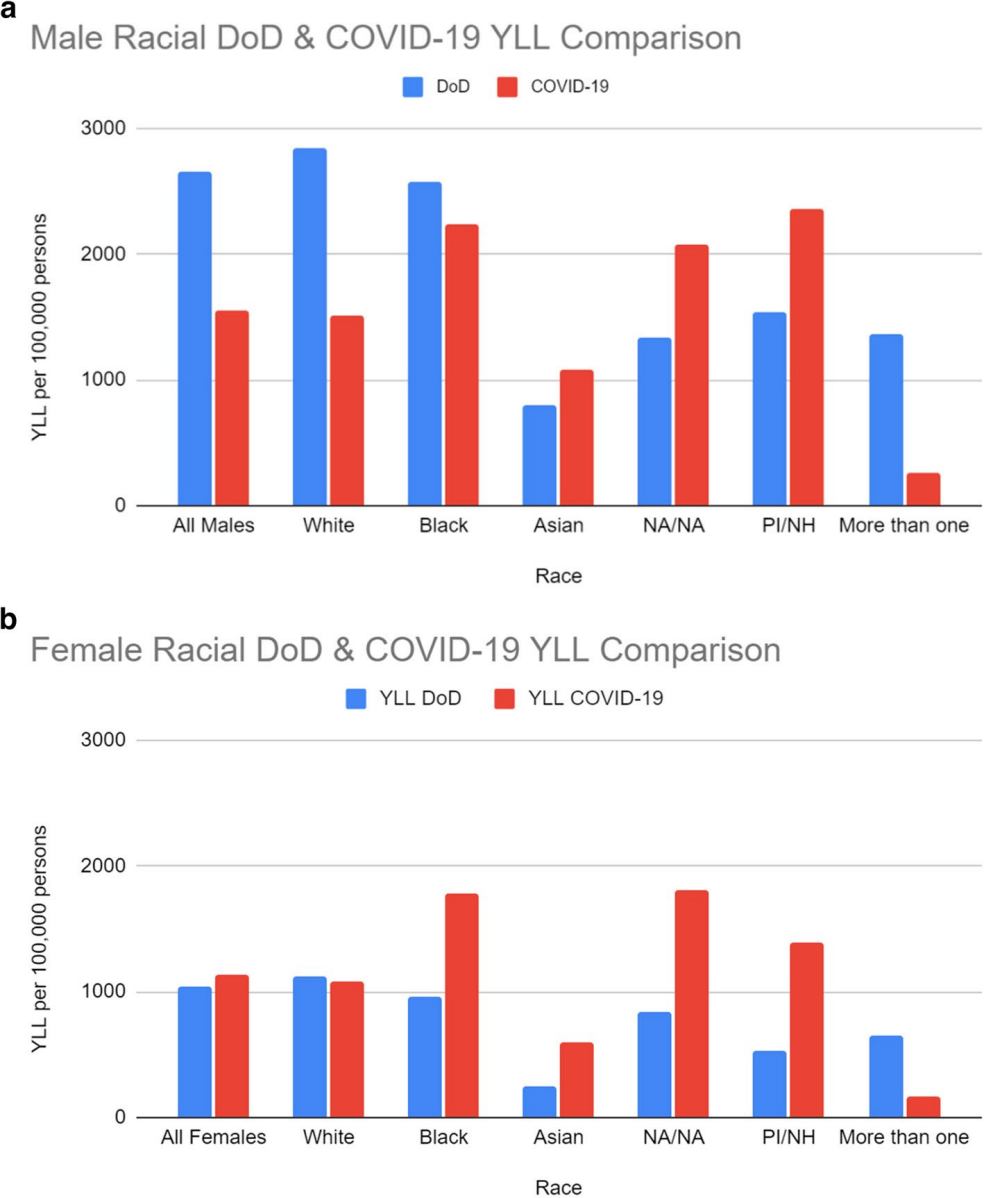
**a** Male racial breakdown of years of life lost Due to COVID-19 and deaths of despair in the US in 2020. **b** Female racial breakdown of years of life lost due to COVID-19 and deaths of despair in the US in 2020

Although fewer men died from deaths of despair (130,801) than COVID-19 (192,512), deaths of despair cost men 71% more YLL than COVID-19. Stated differently, for every 10 years of life men lost due to COVID-19, they lost 17 YLL to deaths of despair. Almost 9 of these YLL were attributable to accidental drug overdose alone. Gender disparities were widest among White, Non-Hispanic or Non-Latino decedents (see Fig. 2). Table 1 presents mortality gender statistics for causes studied.

**Table 1:** Number of deaths, gender years of life lost and mean age at death for COVID-19 and deaths of despair (DoD) including unintentional drug overdose, Alcohol-Induced Causes of Death (AcOD), and suicide in the US in 2020

Cause of Death	Males			Females			Overall		
	**Deaths, No**	**YLL**	**Mean Age (SD)**	**Deaths, No**	**YLL**	**Mean Age (SD)**	**Deaths, No**	**YLL**	**Mean Age (SD)**
COVID-19	192,512	2,515,600.42	73.89 (13.71)	158,319	1,890,098.26	78.73 (13.53)	350,831	4,405,698.68	76.07 (13.65)
DoD	130,801	4,298,945.72	47.25 (16.24)	47,797	1,746,873.29	46.95 (15.14)	178,598	6,045,819.01	47.17 (15.95)
AcOD	59,248	2,186,955.05	42.21 (13.33)	24,310	969,793.83	43.13 (13.50)	83,558	3,156,748.88	42.48 (13.38)
Alcohol Deaths	35,002	884,369.26	56.11 (12.49)	14,059	416,192.69	54.65 (12.56)	49,061	1,300,561.95	55.69 (12.53)
Suicide	36,551	1,227,621.41	46.91 (19.76)	9,428	360,886.77	45.28 (18.13)	45,979	1,588,508.18	46.58 (19.35)

*Abbreviations*: *YLL* Years of life lost, *DoD* Deaths of despair, *AcOD* Accidental drug overdose, *Alcohol Deaths* Alcohol induced causes of death

### Race, COVID-19 and deaths of despair

Racial inequities in mortality burden were observed. Among White, Non-Hispanic or Non-Latino, Americans, deaths of despair (4,935,063.35 YLL) had a substantially higher burden than COVID-19 (3,229,011.85 YLL). Deaths of despair also caused more YLL than COVID-19 among decedents with more than one race. However, for all other racial categories, COVID-19 caused more YLL than deaths of despair. For example, Black Americans lost over one-hundred thousand more years of life to COVID-19 (891,362.53 YLL) than to deaths of despair (771,062.68 YLL).

Population adjusted YLL revealed further disparities. Black Americans proportioned the most YLL per 100,000 at a rate of 2,002 YLL/100 k for COVID-19, compared to just 1,290 for White Americans. Conversely, most deaths of despair YLL were accrued by White Americans with 1,972 YLL/100 k, compared to the 1,226 YLL/100 k average across racial groups. White Americans also had the highest proportion in subcategories for alcohol induced deaths and suicide for YLL/100 k (with 435 and 530 YLL/100 k respectively), but Black Americans had a higher proportion of the subcategory of accidental overdoses, with 1139 YLL/100 k. Table 2 and Fig. 2a and b elaborate on data in context of race and gender.

**Table 2:** Number of deaths, race years of life lost and mean age at death for COVID-19 and deaths of despair including unintentional drug overdose, alcohol-induced causes of death, and suicide in the US in 2020

**Racial Group / CoD**	**YLL**	**YLL per 100 k**	**Incident Deaths**	**ID per 100 k**	**Mean Age (SD)**
***Native American / Native Alaskan***					
COVID-19	83,468.98	1944	4,488	105	67.22 (15.13)
Deaths of Despair	46,798.60	1090	1,148	27	41.26 (13.76)
Unintentional Drug Overdose	41,820.96	974	1,025	24	39.51 (12.11)
Alcohol Induced CoD	972.58	23	33	1	51.98 (14.70)
Suicide	4,005.06	93	90	2	32.29 (14.32)
***Asian***					
COVID-19	165,659.39	828	12,884	64	75.99 (13.62)
Deaths of Despair	102,774.64	514	2,820	14	45.18 (16.66)
Unintentional Drug Overdose	33,594.98	168	844	4	40.26 (15.32)
Alcohol Induced CoD	18,727.10	94	638	3	51.57 (14.41)
Suicide	50,452.56	252	1,338	7	43.72 (19.76)
***Black***					
COVID-19	891,362.53	2002	57,806	130	71.71(14.34)
Deaths of Despair	771,062.68	1732	22,309	50	45.39(14.06)
Unintentional Drug Overdose	507,295.39	1139	14,489	33	45.21(13.79)
Alcohol Induced CoD	116,896.24	263	4,426	10	55.11(11.84)
Suicide	146,871.05	330	3,394	8	35.87(16.21)
***Pacific Islander/Native Hawaiian***					
COVID-19	15,863.43	1880	721	85	62.53(15.49)
Deaths of Despair	8,771.97	1040	219	26	41.54(13.93)
Unintentional Drug Overdose	3,794.33	450	96	11	40.34(12.69)
Alcohol Induced CoD	972.58	115	33	4	51.98(14.70)
Suicide	4,005.06	475	90	11	32.29(14.32)
***White***					
COVID-19	3,229,011.85	1290	273,658	109	77.52 (13.24)
Deaths of Despair	4,935,063.35	1972	147,441	59	48.58 (15.08)
Unintentional Drug Overdose	2,520,547.52	1007	65,938	26	42.30 (13.22)
Alcohol Induced CoD	1,088,444.54	435	41,678	17	55.90 (12.41)
Suicide	1,326,071.29	93	39,825	16	47.55 (18.81)
***More than one race***					
COVID-19	20,332.50	214	1,274	13	71.20 (15.08)
Deaths of Despair	95,859.31	1010	2,305	24	39.84 (13.66)
Unintentional Drug Overdose	49,695.70	523	1,166	12	37.08 (13.42)
Alcohol Induced CoD	13,947.03	147	438	5	49.33 (12.85)
Suicide	32,216.58	339	701	7	33.11 (14.64)

### Ethnicity, COVID-19 and deaths of despair

**Fig. 3:**
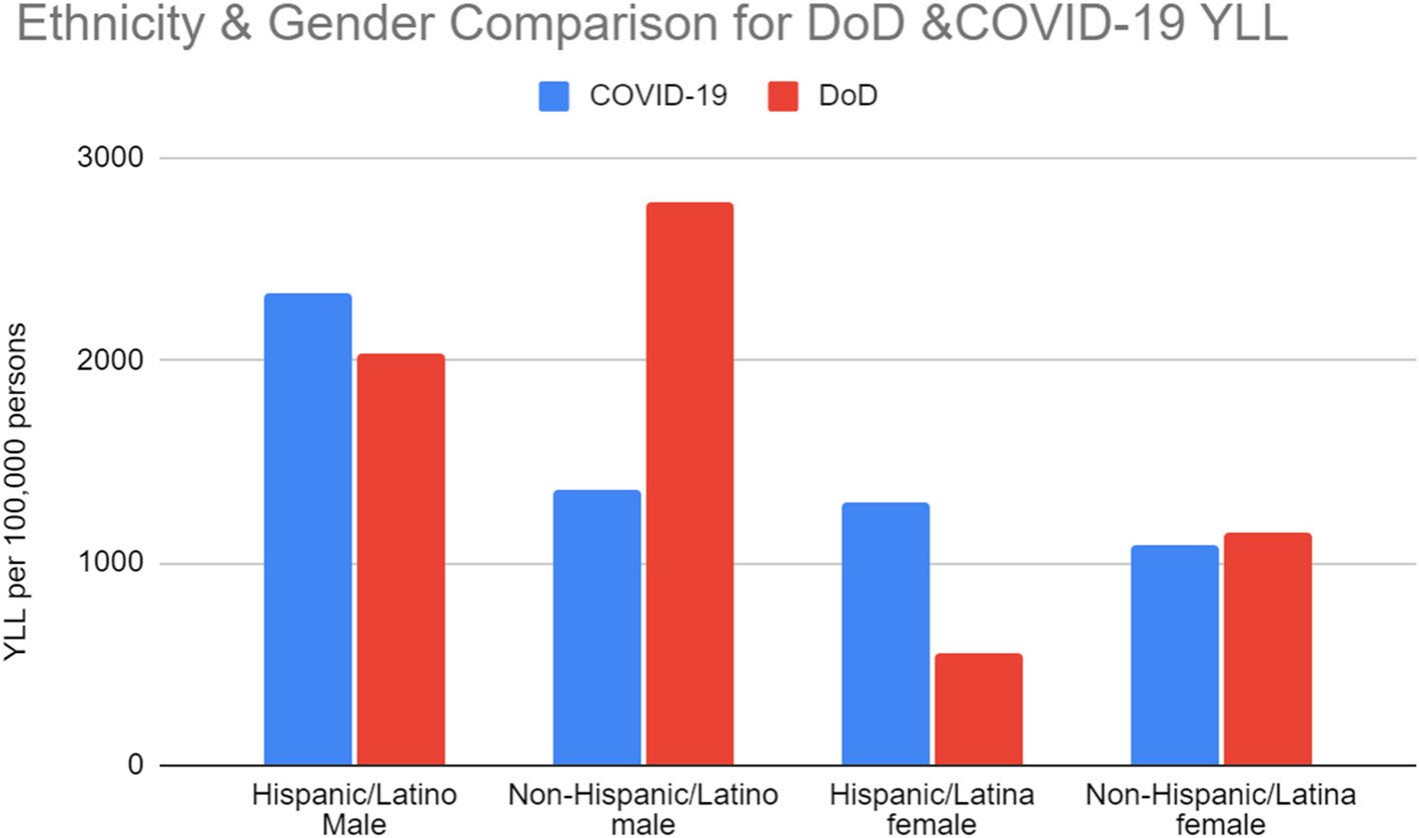
Ethnicity breakdown of Years of Life Lost Due to COVID-19 and deaths of despair in the US in 2020

COVID-19 mortality disproportionately affected Hispanic or Latino Americans, while deaths of despair disproportionately impacted Non-Hispanic or Non-Latino Americans. The mean age of death for these decedents was lower in all categories showing the greater burden of life lost in YLL as well per person. Hispanic or Latino decedents lost 6 more years of life compared to their Non-Hispanic or Non-Latino counterparts to the same cause of death. Both Table 3 and Fig. 3 illustrate more of the ethnicity mortality.

**Table 3:** Number of deaths, ethnicity years of life lost and mean age at death for COVID-19 and deaths of despair in the US in 2020

**Cause of Death**	**Hispanic/Latinx**			**Non-Hispanic/Non-Latinx**		
	**Incident Deaths**	**YLL**	**Mean Age (SD)**	**Incident Deaths**	**YLL**	**Mean Age (SD)**
COVID-19	65,237	1,115,817.84	69.61 (14.81)	284,167	3,269,792.79	77.85 (12.89)
Deaths of despair	21,340	797,311.29	42.34 (14.33)	156,488	5,226,492.59	48.70 (15.15)
Accidental Drug overdose	10,032	406,013.15	39.22 (13.37)	73,035	2,736,326.55	43.05 (13.36)
Alcohol-induced causes of death	6,737	195,273.10	52.16 (13.17)	42,157	1,101,112.61	55.86 (12.39)
Suicide	4,571	196,025.04	35.66 (16.24)	41,296	1,389,053.43	47.20 (18.90)

*Abbreviations*: *YLL* Years of life lost

## Discussion

The COVID-19 pandemic brought immense loss of life, and the deaths of despair at the time created nearly 40% more YLL than COVID-19 alone in the US in 2020. There was a larger burden due to deaths of despair with 6,045,819.01 YLL compared to COVID-19 4,405,698.68 YLL. While much of this burden occurred in the White population, racial and ethnic minority populations had disproportionate mortality of COVID-19, while still being impacted by deaths of despair. The COVID-19 pandemic aggravated the gaps in national involvement and preparedness for an infectious disease response, as well as elucidated the need for greater investment in behavioral health needs of the nation, and its underserved populations [13, 14].

Deaths of despair decedents were younger than COVID-19 decedents, losing disproportionately more years of work, community, and family life. Similarly, men experienced more YLL than women, particularly for deaths of despair, despite shorter average lifespan. In contrast, racial and ethnic minorities were greatly impacted by COVID-19 associated mortality, alongside great burden of deaths of despair YLL. It has been shown how overdoses during the pandemic also disproportionately affected Black, Latino, and Native American populations [3]. It is important to frame this data in the context of time insofar as it represents only the onset of the COVID-19 pandemic, using data for the year 2020. Further analysis is needed to understand the effects of COVID-19 and pandemic related deaths of despair long-term.

While population adjusted YLL showed a greater burden of deaths of despair YLL amongst racial and ethnic minorities, it did not outnumber the deaths of despair YLL among White, Non-Hispanic or Non-Latino Americans. This could be due to the differing barriers faced between the two groups. Often racial and ethnic minorities are forced to continually build up resilience when navigating extra barriers such as racism and maneuvering through a dominant and nonidentical American culture [15]. The capacity to withstand and recover quickly from frequent obstacles as such could be a contributing factor to a lower deaths of despair YLL in racial and ethnic minorities.

Conversely, Black Americans had the greatest proportion of accidental overdoses in deaths of despair YLL. This speaks to the lack of substance use treatment options tailored to the unique needs of Black Americans. Black Americans face multiple layers of racism that impact readiness, initiation and engagement of substance use treatment [16, 17]. Examples include difficulties in geographic access to substance use treatment due to redlining, lower rates of insurance, and implementation of criminal punishments rather than treatment referral [18]. To change the trajectory of accidental overdose deaths for Black Americans, it is necessary to create culturally responsive, easily accessible substance use treatment options given the historic and current racial discrimination this population encounters [19, 20].

Comorbid deaths show the overlap of burden between COVID-19 and deaths of despair, and how the two are not mutually exclusive. Yet, deaths of despair have not received funding comparable to the COVID-19 response. It is quite plausible that the observed deaths would be mitigated with increased funding for behavioral health and addiction medicine, and increased access to culturally informed care for historically disadvantaged groups. These deaths might have been absent without the comorbidity of COVID-19 or cooccurrence of stress-inducing pandemic circumstances, such as increased isolation, decreased social support, and maladaptive coping with substance use. Of deaths of despair, accidental drug overdose was the most burdensome, causing nearly three quarters as many YLL as COVID-19; COVID-19 pandemic had intense repercussions on deaths of despair especially overdoses with increases in overdose deaths seen in 2020 [3].

Deaths of despair are seen to clearly impact younger populations, and our data takes it a step further to understand the extent to which COVID-19 and deaths of despair impact people from intersecting identities, such as those who are both historically excluded due to race and class (e.g., Black women from a low socioeconomic status) [21]. For instance, one study demonstrated how racial and ethnic minorities were more likely to experience high fear of COVID-19 infection and perceived threat to health from the disease [22]. These perceptions and the associated fear response should be predictive of countermeasures and behaviors to protect oneself, including hand washing and mask wearing, thereby reducing disease burden and mortality. Nevertheless, the analyzed data did not express the protective benefits expected of these behaviors, and other variables impacting racial and ethnic minorities likely outweighed these benefits [23–25]. Conversely, these attitudes were found in females more often than males, consistent with observed gender mortality differences [22]. This inequality could be accounted for by extenuating medical discrimination and structural racism impacting racial and ethnic minoritized groups. For example, in one health systems study of hospitalized COVID-19 patients in the initial weeks of the pandemic, 76% were Black; similarly, of those who died in this sample, 70% were Black [26]. This could reflect structural racism impacting Black communities, by creating disparities in health outcomes due to limited access, inadequate housing, and poverty increasing vulnerability to complications of the virus. Discrimination and bias in healthcare delivery can lead to delayed diagnosis, inadequate treatment, and poor outcomes [27].

The impact of the COVID-19 pandemic on the world has been immense and experienced by all. Research reported by Kramer showed how more YLL were lost to COVID-19 than accidents alone in the US; and that only heart disease and cancer had greater YLL impacts than COVID-19 [28]. The present work confirms and builds off these findings to see the comparison with deaths of despair. Deaths of despair were spurred by the pandemic inhibiting access to care and increasing isolation and distress leading to negative reinforcement coping [29]. Coping mechanisms like increased substance use were supported by increased purchasing and consumption of alcohol because of lockdown and the pandemic’s stressors [30].

Suicide’s contribution to deaths of despair could be connected to trends in prior pandemics and epidemics. For example, studies reported increases suicide rates during the 1918–1919 Influenza Pandemic and the Hong Kong SARS epidemic [31, 32]. Individuals that suffer from psychiatric disorders may experience exacerbation in their condition related to the stressors, such as those associated with the COVID-19 pandemic in 2020. Such exacerbation might increase the risk of suicide, further contributing to deaths of despair. We propose that to mitigate this, there should be a corresponding infusion towards promoting more robust behavioral health education and sources of support [33]. Furthermore, corollary effects of the pandemic such as unemployment and social distancing can contribute to stress and problems leading to Deaths of Despair [14, 34].

This study analyzes the death across race, gender and ethnicity in a turbulent time; yet it does so with limitations. The study period includes January when no COVID-19 deaths occurred. Also, the dataset did was not able to control for confounds such as access to healthcare. This dataset was compiled using the CDC WONDER Database, which inherently has limitations as it procures data from death records which can lead to undercounting, misclassification, and human error. A CDC report showed how misclassification in death statistics most greatly affects Native American and Native Alaskan populations in undercounting and misclassification. The same study showed how Asian, Hispanic, and Native Hawaiian/Pacific Islander groups were also affected but not to the same degree as Native American and Native Alaskan groups [35]. Other literature supports the efficacy of using the current YLL approach in reporting important mortality statistics [8, 36]. Although limited by death records which may potentially misclassify deaths of despair and COVID-19, our findings show deaths of despair are a severe and preventable mortality burden in the US, associated with YLL comparable in scale to COVID-19. An assiduous public health response to deaths of despair is needed during the ongoing COVID-19 pandemic, and beyond.

During the COVID-19 pandemic, the US has mounted a required and historically unprecedented public health response; with federal investment greater than $3.5 trillion, including billions toward life-saving research, treatment, and prevention [37]. A clear implication of the present study is that deaths of despair deserve a similarly historic societal effort, alongside COVID-19. Unfortunately, only 1 in 10 individuals with substance use disorders and 4 in 10 people needing mental health care could access treatment during 2020 [38]. Therefore, we join prior calls for a national resilience strategy to mitigate deaths of despair [39]. Such a strategy should bolster behavioral health research, support mental health workforce development and education, increase access to evidence-based substance use treatment and address systemic inequities and social determinants of deaths of despair.

## Data Availability

Not Applicable.

## Abbreviations

YLL: Years of Life Lost
CDC WONDER: Centers for Disease Control Wide-Ranging Online Data for Epidemiologic Research
